# Triglyceride-glucose index and cancer risk: a prospective cohort study in Taiwan

**DOI:** 10.1186/s13098-025-01768-8

**Published:** 2025-07-18

**Authors:** Hsiao-Yun Yeh, Mei-Hung Pan, Chih-Jen Huang, Shiao-Ya Hong, Hwai-I Yang, Ying-Ying Yang, Chia-Chang Huang, Hung-Cheng Tsai, Tzu-Hao Li, Chien-Wei Su, Ming-Chih Hou

**Affiliations:** 1 Department of Medical Education, Taipei Veterans General Hospital, No. 201, Sec. 2, Shih-Pai Road, 11217 Taipei, Taiwan; 2 School of medicine, National Yang Ming Chiao Tung University, Taipei, Taiwan; 3 Department of Family Medicine, Taipei Veterans General Hospital, Taipei, Taiwan; 4 Genomics Research Center, Academia Sinica, 115, Taipei, Taiwan; 5 Department of Biotechnology and Laboratory Science in Medicine, National Yang Ming Chiao Tung University, Taipei, Taiwan; 6 Institute of Clinical Medicine, National Yang Ming Chiao Tung University, Taipei, Taiwan; 7 Graduate Institute of Medicine, College of Medicine, Kaohsiung Medical University, Kaohsiung, Taiwan; 8 Biomedical Translation Research Center, Academia Sinica, Taipei, Taiwan; 9 Department of Public Health, China Medical University, Taichung, Taiwan; 10 Division of Gastroenterology and Hepatology, Department of Medicine, Taipei Veterans General Hospital, Taipei, Taiwan; 11 Division of Endocrinology and Metabolism, Department of Medicine, Taipei Veterans General Hospital, Taipei, Taiwan; 12 Division of Allergy and Immunology, Department of Medicine, Taipei Veterans General Hospital, Taipei, Taiwan; 13 Division of Allergy and Immunology, Department of Internal Medicine, Shin Kong Wu Ho-Su Memorial Hospital, Taipei, Taiwan; 14 School of Medicine, College of Medicine, Fu Jen Catholic University, Taipei, Taiwan; 15 Division of General Medicine, Department of Medicine, Taipei Veterans General Hospital, Taipei, Taiwan

## Abstract

**Background:**

Insulin resistance (IR) is a key metabolic abnormality associated with adverse health outcomes, including increased cancer risk. The triglyceride-glucose (TyG) index, a validated surrogate marker of IR, has been linked to metabolic dysfunction; however, its association with cancer risk in large population-based cohorts remains unclear. This study aimed to evaluate the relationship between TyG index and cancer risk in Taiwanese population.

**Methods:**

We analyzed 150,592 participants from the Taiwan Biobank, among whom 148,809 were linked to the Taiwan Cancer Registry (2011–2022) for cancer incidence tracking. Cancer risk was assessed across TyG quartiles over a median follow-up of 5.7 years (IQR: 3.4–7.6). Hazard ratios (HRs) and 95% confidence intervals (CIs) were estimated using Cox proportional hazards models, adjusting for key covariates.

**Results:**

Higher TyG index levels were associated with increased risks of digestive system cancer (adjusted HR [aHR]: 1.17, 95% CI: 1.05–1.29), colorectal cancer (aHR: 1.25, 95% CI: 1.08–1.44), and urinary tract cancer (aHR: 1.47, 95% CI: 1.18–1.85). While subgroup trends suggested numerically higher risks in males, individuals aged ≥ 50 years, and those with overweight or obesity for these cancer types, formal interaction tests did not support statistically significant effect modification in these groups. Significant interactions were observed for overall cancers by age (*P* < 0.001) and BMI (*P* = 0.012), and for urinary tract cancer by drinking status (*P* = 0.047). In a subset of 19,808 participants with follow-up data, higher TyG quartiles were also linked to fatty liver, carotid plaques, and persistent IR over time (*r* = 0.75).

**Conclusions:**

Higher TyG index levels, indicative of greater IR, are associated with an elevated risk of digestive system, colorectal, and urinary tract cancers. Evaluating TyG index levels could assist in risk stratification for these cancers among individuals with persistent IR, supporting targeted prevention strategies.

**Supplementary information:**

The online version contains supplementary material available at 10.1186/s13098-025-01768-8.

## Introduction

Insulin resistance (IR) is a pathological condition characterized by a diminished cellular response to insulin, requiring higher insulin levels to maintain normal glucose homeostasis [1]. IR plays a crucial role in linking obesity and metabolic syndrome (MetS) to a range of adverse health outcomes, including diabetes, cardiovascular diseases, and various cancers [2–5].

Excess body weight was estimated to account for approximately 3.9% (544,300 cases) of all cancer cases globally in 2012 [6]. MetS [7], characterized by central obesity, dyslipidemia, hypertension, and IR, has also been linked to an increased risk of malignancies such as colorectal, breast, and liver cancer [8, 9]. With the rising prevalence of obesity and MetS, alongside the increasing global cancer burden, there is a growing need to better characterize the relationship between IR and cancer risk using reliable markers, facilitating broad population-based research and enabling early targeted risk assessment and prevention strategies.

IR induces compensatory hyperinsulinemia, which promotes tumorigenesis by stimulating cell proliferation and inhibiting apoptosis through multiple signaling pathways [4, 10]. Epidemiological studies have reported an increased risk of several cancers, such as breast, colorectal, liver, pancreatic, endometrial, lung, and prostate cancers, are higher in patients with IR [10]. Traditionally, IR has been measured using methods such as the hyperinsulinemic-euglycemic clamp or the homeostasis model assessment of IR (HOMA-IR) [11, 12]. However, these methods can be complex, costly, and impractical for large-scale epidemiological studies. The triglyceride-glucose (TyG) index, derived from fasting triglyceride (TG) and glucose levels, has been validated as a reliable surrogate marker of IR [12–14]. Due to its simplicity and accessibility, the TyG index is particularly useful for large population-based studies [15, 16].

While the TyG index has been increasingly recognized as a predictor of metabolic disorders, including T2DM, cardiovascular disease, and stroke, its role in cancer risk remains underexplored [17–20]. To address this gap, we conducted a retrospective longitudinal cohort study to examine the association between IR, measured by TyG index, and long-term cancer risk, considering potential interactions with lifestyle factors such as smoking, alcohol drinking and exercise. Additionally, we examined the anthropometric and metabolic profiles of individuals across different TyG index ranges. Through this approach, we aim to identify cancers strongly associated with IR and, by utilizing reliable markers such as the TyG index, improve risk stratification and facilitate the development of personalized prevention and treatment strategies.

## Materials and methods

### Data source and study population

This study utilized data from the Taiwan Biobank (TWB), a government-supported database established in 2012, to integrate genomic, lifestyle, dietary, and environmental exposure information for epidemiologic and biomedical research in the Taiwanese population. By December 2021, TWB included 150,709 adults aged 30 to 70 years with no self-reported cancer diagnosis. All participants provided written informed consent for data and sample collection and anonymized data sharing. Baseline data were collected through questionnaires, physical examinations, and blood and urine tests. Follow-up visits, initiated in 2016 and occurring 2–4 years after baseline, included repeat measurements and additional imaging studies such as abdominal ultrasonography and dual-energy X-ray absorptiometry. These standardized data collection methods have been detailed in prior studies [21, 22].

A significant strength of TWB is its linkage to Taiwan’s National Health Insurance Research Database (NHIRD) and the Taiwan Cancer Registry (TCR), providing nearly complete coverage of participants’ lifelong health records. The TCR, established in 1979, collects nationwide data on newly diagnosed cancers and has included detailed information on staging, treatment, and recurrence since 2002 [23]. For this study, cancer incidence was tracked through linkage with Taiwan Cancer Registry data, with follow-up continuing through December 31, 2022. Analyses were conducted with authorized access at the Data Science Center, Ministry of Health and Welfare. This study was conducted in accordance with the Declaration of Helsinki and was approved by the Institutional Ethical Review Board of Academia Sinica (AS-IRB-BM-16015).

### Study design

This analysis was based on prospectively collected data from the Taiwan Biobank cohort. We assessed demographic, lifestyle, anthropometric, and metabolic profiles associated with IR using the TyG index as the primary measure and evaluated its association with the incidence of various cancer types.

The TyG index was calculated using the formula: ln [fasting blood TG (mg/dl) × fasting blood glucose (mg/dl)/2] [24]. Participants lacking TG or glucose data were excluded from the analysis. The remaining participants were divided into quartiles (Q1–Q4) based on their TyG index values.

**Fig. 1:**
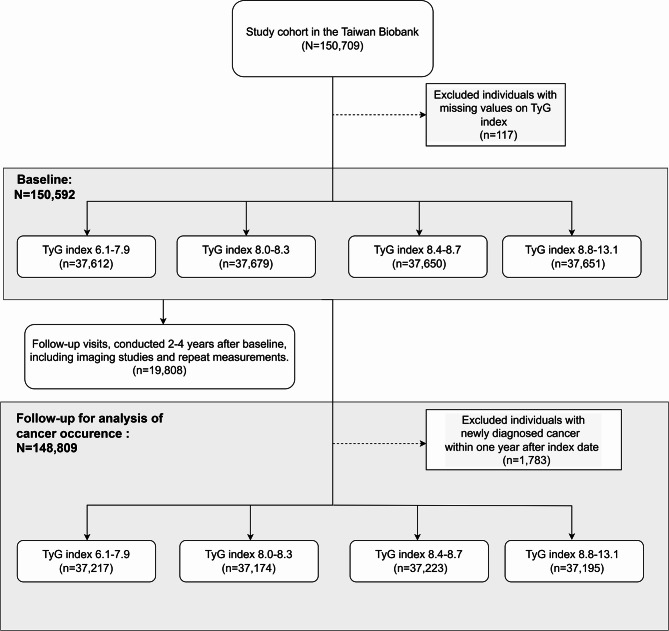
Study flowchart: participant selection and TyG index distribution. Flowchart depicting the selection of participants from the Taiwan Biobank (N = 150,709) for the study. A total of 150,592 participants with complete TyG index data at baseline were included, after excluding 117 individuals with missing TyG data. Participants were categorized into four TyG index quartiles. Additionally, 19,808 individuals underwent abdominal and carotid ultrasounds at follow-up visits. For cancer incidence analysis, 148,809 participants were included after excluding 1,783 individuals diagnosed with cancer within one year of enrollment. Follow-up cancer incidence was analyzed based on TyG index quartiles

To reduce prevalent bias, focus on incident cases, and account for cancer latency, individuals diagnosed with cancer within the first year of enrollment were excluded. A flowchart depicting the sample selection process, exclusion criteria, TyG and TyG index quartile classification is provided in Fig. 1. Follow-up visits, conducted 2–4 years after baseline, included repeat measurements and imaging studies, such as liver and carotid ultrasounds. Participants who completed the follow-up assessments were reclassified into TyG quartiles based on the follow-up measurements, enabling analysis of the association between TyG index and metabolic outcomes over time.

### Baseline data collection

At study entry, we collected comprehensive data to investigate characteristics across TyG index quartiles. Variables included demographic factors (sex, age), and anthropometric measurements (height, weight, waist circumference [WC], hip circumference [HC], systolic and diastolic blood pressure [SBP and DBP], and body fat percentage). Body mass index (BMI) was calculated as weight (kg) divided by height squared (m²) and categorized according to Taiwan’s Ministry of Health and Welfare guidelines: normal weight (BMI < 24), overweight (BMI 24.0–26.9), and obesity (BMI ≥ 27.0). Smoking status was defined as current smoking if participants had smoked continuously for at least six months. Current drinking was defined as consuming at least 150 cc of alcohol per week for the past six months. Regular exercise was defined as engaging in physical activity at least three times per week for 30 min or more [25–27].

Metabolic blood biochemistry data included total cholesterol (TC), TG, high-density lipoprotein cholesterol (HDL-C), low-density lipoprotein cholesterol (LDL-C), fasting blood glucose (FPG), hemoglobin A1c (HbA1c), alanine aminotransferase (ALT), aspartate aminotransferase (AST), and uric acid (UA).

### MetS definition and adiposity index calculations

MetS in this study was defined based on the 2007 guidelines issued by Taiwan’s Ministry of Health and Welfare, which were adapted from the 2005 International Diabetes Federation (IDF) criteria [7]. The adapted criteria incorporate modifications specific to Taiwan’s population while maintaining alignment with international definitions. The components of MetS include abdominal obesity (waist circumference ≥ 90 cm in men or ≥ 80 cm in women), elevated TG (≥ 150 mg/dL), reduced HDL-C (< 40 mg/dL in men or < 50 mg/dL in women), elevated blood pressure (SBP ≥ 130 mmHg or DBP ≥ 85 mmHg) and elevated fasting glucose (≥ 100 mg/dL). Individuals were classified as having MetS if they met three or more of these criteria. We calculated the proportion of individuals with MetS and its components across TyG index quartiles. To assess adiposity-related measures, we derived several indices from anthropometric and biochemical data, including body adiposity index (BAI), visceral adiposity index (VAI), waist-to-hip ratio (WHR), and the triglyceride to HDL-C ratio (THR) [28–34].

The BAI, an estimate of body fat percentage, was calculated as: BAI = [HC (cm) / height (m)^1.5^] – 18 [31]. The VAI, calculated differently for men and women, incorporated WC, BMI, TG, and HDL to estimate visceral fat accumulation [32]. The WHR, calculated as WC divided by HC, assessed abdominal fat distribution [33]. The THR, calculated as TG divided by HDL-C, provided additional insight into lipid profiles [34].

### Longitudinal follow-up and medical imaging: correlation of TyG index over time

At follow-up visits, participants underwent repeat measurements of biochemical parameters and additional imaging studies, including liver and carotid ultrasounds, to assess the presence and severity of fatty liver and carotid plaques. We compared the prevalence of these conditions across TyG index quartiles at follow-up. Additionally, we analyzed the correlation between baseline and follow-up TyG index levels to evaluate the consistency of individuals’ IR status over time.

### Incidence of cancers associated with TyG index quartiles

After excluding participants diagnosed with cancer within the first year of enrollment, we tracked the remaining cancer-free individuals for new cancer diagnoses via the TCR until December 2022. Cancer types were classified according to ICD-9 codes 140–194 and ICD-10 codes C00-C75.

#### Cancer screening by trend analysis

We extracted data on newly diagnosed cancers, including type and date of diagnosis, from the TCR. Trend analyses were performed to identify cancers with increasing incidence across higher TyG quartiles.

#### Survival analysis

Kaplan-Meier survival curves were used to assess cumulative cancer incidence for selected cancers, stratified by TyG quartiles, allowing evaluation of long-term cancer risk based on baseline TyG quartiles.

#### Adjusted and stratified analysis

We performed adjustment and stratified analysis based on sex, age, BMI, smoking, drinking, and exercise habits, to explore the independent associations between TyG index and cancer risk across different subgroups. Hazard ratios (HRs) for specific cancers were calculated using the TyG index as a continuous variable. In addition, we assessed potential effect modification by incorporating multiplicative interaction terms between the TyG index and key covariates into Cox regression models.

### Statistical analysis

We presented categorical variables as percentages and continuous variables as means with standard deviations (SD). Differences across TyG index quartiles were assessed using Chi-square tests for categorical variables and one-way ANOVA for continuous variables. Cox proportional hazards regression models were used to estimate HRs and 95% confidence intervals (CIs) for cancer incidence with adjustment for potential confounders such as sex, age, BMI, smoking, drinking, and exercise habits. We used P_trend_ to assess risk trends across TyG quartiles. False discovery rate (FDR) correction was applied to control for multiple comparisons in the cancer incidence analysis. Kaplan-Meier survival analysis estimated cumulative cancer incidence rates for specific cancers, with differences between quartiles evaluated using the log-rank test. In stratified analyses, HRs adjusted for sex, age, BMI, smoking, drinking, and exercise quantified the effect of each unit increase in TyG index on cancer risk within subgroups. Statistical analyses were performed using R version 4.3.2 (RRID: SCR_001905) and Stata version 14 (RRID: SCR_012763). A two-tailed significance level was set at *p* < 0.05 for all analyses.

## Results

### Baseline demographic, lifestyle, and metabolic profiles across TyG index quartiles

A total of 150,592 participants (36.3% men) were included. Baseline characteristics stratified by TyG index quartiles are detailed in Table 1, including demographic factors (age, sex), lifestyle habits (smoking, drinking, exercise), anthropometrics (BMI, body fat percentage, BAI, VAI, WHR), and metabolic parameters (MetS components and biochemical markers).

**Table 1:** Baseline characteristics across TyG index quartiles in the Taiwan biobank population

Baseline variables^a^	Total	TyG index^b^, Q1 (6.1–7.9)	TyG index, Q2 (8.0-8.3)	TyG index, Q3 (8.4–8.7)	TyG index, Q4 (8.8–13.1)	*P* value^c^	*P* value^c^ (adjusted for age and sex)
	*N* = 150,592	*N* = 37,612	*N* = 37,679	*N* = 37,650	*N* = 37,651		
Men	54,670 (36.3)	7,936 (21.1)	12,043 (32.0)	15,281 (40.6)	19,410 (51.6)	< 0.001	-
Age, yr	49.45 (11.4)	45.04 (11.3)	49.46 (11.4)	51.34 (11.0)	51.93 (10.6)	< 0.001	-
Smoking^d^	13,616 (9.0)	1,906 (5.1)	2,729 (7.3)	3,679 (9.8)	5,302 (14.1)	< 0.001	< 0.001
Drinking^e^	9,147 (6.1)	1,401 (3.7)	1,853 (4.9)	2,350 (6.2)	3,543 (9.4)	< 0.001	< 0.001
Exercise^f^	58,295 (38.7)	13,761 (36.6)	15,291 (40.7)	15,182 (40.3)	14,061 (37.3)	< 0.001	< 0.001
Metabolic syndrome	44,676 (29.7)	1,807 (5.0)	5,409 (14.4)	11,247 (29.9)	31,113 (82.6)	< 0.001	< 0.001
Abdominal obesity^g^	70,463 (46.8)	9,635 (25.6)	15,392 (40.9)	20,289 (53.9)	25,147 (66.8)	< 0.001	< 0.001
High blood pressure^h^	47,897 (31.8)	5,776 (15.4)	10,195 (27.1)	13,755 (36.5)	18,171 (48.3)	< 0.001	< 0.001
High fasting glucose^i^	32,042 (21.3)	1,808 (5.0)	4,897 (13.0)	8,810 (23.4)	16,527 (43.9)	< 0.001	< 0.001
High triglyceride^j^	31,518 (20.9)	0 (0.0)	0(0.0)	843 (2.2)	30,675 (81.5)	< 0.001	< 0.001
Low HDL-C^k^	37,225 (24.7)	2,908 (7.7)	5,621 (14.9)	9,929 (26.4)	18,767 (49.8)	< 0.001	< 0.001
BMI, kg/m^2^	24.3 (3.9)	22.1 (3.0)	23.6 (3.5)	24.9 (3.7)	26.4 (3.9)	< 0.001	< 0.001
Body fat rate, %	28.7 (7.5)	26.7 (6.8)	28.3 (7.3)	29.6 (7.6)	30.5 (7.6)	< 0.001	< 0.001
BAI	21.6 (3.1)	20.7 (2.7)	21.4 (3.0)	21.9 (3.1)	22.3 (3.2)	< 0.001	< 0.001
VAI	1.7 (1.9)	0.6 (0.2)	1.0 (0.3)	1.6 (0.5)	3.6 (3.1)	< 0.001	< 0.001
WHR	0.87 (0.07)	0.82 (0.06)	0.85 (0.06)	0.88 (0.06)	0.91 (0.06)	< 0.001	< 0.001
HbA1, %	5.8 (0.8)	5.2 (0.3)	5.6 (0.4)	5.8 (0.6)	6.2 (1.3)	< 0.001	< 0.001
Fasting glucose, mg/dL	96.0 (20.6)	88.2 (6.9)	92.1 (8.6)	95.6 (12.1)	108.3 (34.7)	< 0.001	< 0.001
Total cholesterol, mg/dL	195.8 (37.0)	183.6 (32.5)	193.0 (37.7)	199.6 (34.9)	206.9 (38.7)	< 0.001	< 0.001
Triglyceride, mg/dL	115.7 (93.8)	51.4 (10.1)	79.0 (10.4)	113.9 (17.5)	218.4 (136.7)	< 0.001	< 0.001
HDL-C, mg/dL	54.9 (13.6)	64.0 (13.1)	57.9 (12.3)	52.2 (11.2)	45.2 (9.7)	< 0.001	< 0.001
LDL-C, mg/dL	120.8 (31.9)	107.1 (27.4)	120.4 (29.4)	128.7 (31.1)	127.1 (34.7)	< 0.001	< 0.001
Albumin, g/dL	4.5 (0.2)	4.5 (0.2)	4.5 (0.2)	4.5 (0.2)	4.6 (0.2)	< 0.001	< 0.001
AST, U/L	24.6 (13.0)	22.5 (11.1)	23.6 (14.0)	25.8 (12.1)	27.4 (13.8)	< 0.001	< 0.001
ALT, U/L	24.1 (20.9)	17.92 (15.7)	21.21 (20.1)	25.03 (19.6)	32.2 (24.6)	< 0.001	< 0.001
Creatinine, mg/dL	0.7 (0.3)	0.7 (0.3)	0.7 (0.3)	0.7 (0.3)	0.8 (0.4)	< 0.001	< 0.001
Uric acid, mg/dL	5.4 (1.4)	4.7 (1.2)	5.2 (1.3)	5.6 (1.4)	6.1 (1.5)	< 0.001	< 0.001
THR	2.5 (2.9)	0.8 (0.3)	1.4 (0.4)	2.3 (0.6)	5.2 (4.7)	< 0.001	< 0.001

^a^ Data are presented as number (%) for categorical variables or mean (SD) for numerical variables. WHR is reported to two decimal places to maintain differentiation across quartiles that would be lost with single-digit rounding. ^b^ TyG index calculated as: ln[triglycerides(mg/dl) x blood glucose(mg/dl)/2]. Participants were divided into quartiles (Q1–Q4) based on their TyG index levels. ^c^ Differences were tested with chi-square for categorical variables and with analysis of covariance for numerical data. ^d^ Smoking: defined as current smokers, having smoked continuously for 6 months and being still smoking at the time of the interview. ^e^ Drinking: defined as having a drinking habit (150 c.c/week for 6 months) and being currently drinking alcohol. ^f^ Exercise: defined as exercising regularly (at least three times a week for 30 min or more). ^g^ Abdominal Obesity: WC ≥  90 cm in men; ≥ 80 cm in women. ^h^ High blood pressure: SBP ≥  130mmHg or DBP ≥  85mmHg. ^i^ High fasting glucose: fasting glucose ≥  100 mg/dL. ^j^ High triglyceride: triglyceride ≥  150 mg/dL. ^k^ Low HDL-C: refer to high density lipoprotein cholesterol (HDL-C) <  40 mg/dL in men; <50 mg/dL in women.Abbreviations: TyG Index, triglyceride-glucose index; BMI, body mass index; BAI, body adiposity index; VAI, visceral adiposity index; WHR, waist-to-hip ratio; HbA1c, hemoglobin A1c; HDL-C, high-density lipoprotein cholesterol; LDL-C, low-density lipoprotein cholesterol; AST, aspartate aminotransferase; ALT, alanine aminotransferase; THR, triglyceride to HDL-C ratio

Higher TyG quartiles were consistently associated with adverse metabolic profiles. The proportion of men, smokers, and drinkers increased significantly across quartiles (*p* < 0.001). Age also increased progressively, from 45.0 years in the lowest quartile to 51.9 years in the highest (*p* < 0.001). Regular exercise peaked in the middle quartiles but slightly decreased in the highest quartile.

Metabolically, higher TyG quartiles exhibited worsened profiles. The prevalence of MetS rose from 5.0% in Q1 to 82.6% in Q4 (*p* < 0.001), with increased rates of abdominal obesity, hypertension, hyperglycemia, and dyslipidemia. Glycemic markers (HbA1c, fasting glucose) rose, while HDL-C levels declined (*p* < 0.001). Anthropometric measures, liver enzymes, and uric acid levels also showed significant increases, indicating worsening metabolic function.

### Demographic, lifestyle, and metabolic characteristics at follow-up and association with fatty liver and carotid plaques

At a median follow-up of 3.8 (IQR: 3.8–4.6) years, 19,808 participants completed follow-up visits, which included ultrasound assessments for fatty liver and carotid plaques. Metabolic blood biochemistry data at follow-up visits were also measured. The metabolic trends observed at baseline persisted, with higher TyG quartiles consistently linked to worse profiles. The prevalence of moderate or severe fatty liver diagnosed by abdominal ultrasonography increased from 2.8% in Q1 to 55.8% in Q4, while moderate or severe carotid plaques rose from 6.6 to 14.9% (*p* < 0.001) (Table 2). The strong correlation between baseline and follow-up TyG indices (*r* = 0.75) (Supplementary Fig. 1) demonstrates the persistence of IR over time. This finding supports the use of the TyG index as a reliable marker for tracking long-term metabolic risk and monitoring adverse metabolic outcomes throughout the follow-up period.

**Table 2:** Characteristics across TyG index quartiles in participants with follow-up sonography from the Taiwan Biobank

Follow-up variables^a^	Total	TyG index^b^, Q1 (6.7-8.0)	TyG index, Q2 (8.1–8.4)	TyG index, Q3 (8.5–8.8)	TyG index, Q4 (8.9–13.7)	P value^c^	P value^c^ (adjusted for age and sex)
	*N* = 19,808	*N* = 4,960	*N* = 4,949	*N* = 4,949	*N* = 4,950		
Men	7,072 (35.7)	1,167 (23.5)	1,637 (33.1)	1,971 (39.8)	2,297 (46.4)	< 0.001	-
Age, yr	55.45 (10.3)	52.58 (10.9)	55.70 (10.1)	56.7 (9.7)	56.8 (9.6)	< 0.001	-
Smoking^d^	1,560 (7.9)	193 (3.9)	310 (6.3)	409 (8.3)	648 (13.1)	< 0.001	< 0.001
Drinking^e^	1,129 (5.7)	196 (4.0)	244 (4.9)	269 (5.4)	420 (8.5)	< 0.001	< 0.001
Exercise^f^	8,913 (45.0)	2,150 (43.3)	2,319 (46.9)	2,328 (47.0)	2,116 (42.7)	< 0.001	< 0.001
Metabolic syndrome	5,059 (25.5)	128 (2.6)	415 (8.4)	1,017 (20.5)	3,499 (70.7)	< 0.001	< 0.001
Abdominal obesity^g^	9,853 (49.7)	1,525 (30.7)	2,211 (44.7)	2,774 (56.1)	3,343 (67.5)	< 0.001	< 0.001
High blood pressure^h^	8,080 (40.8)	1,249 (25.2)	1,817 (36.7)	2,282 (46.1)	2,732 (55.2)	< 0.001	< 0.001
High fasting glucose^i^	4,974 (25.1)	378 (7.6)	829 (16.8)	1,374 (27.8)	2,393 (48.3)	< 0.001	< 0.001
High triglyceride^j^	4,537 (22.9)	0 (0.0)	0 (0.0)	295 (6.0)	4,242 (85.7)	< 0.001	< 0.001
Low HDL-Ck	5,143 (26.0)	367 (7.4)	774 (15.6)	1,360 (27.5)	2,644 (53.4)	< 0.001	< 0.001
BMI, kg/m^2^	24.3 (3.7)	22.4 (3.0)	23.8 (3.3)	25.0 (3.6)	26.2 (3.7)	< 0.001	< 0.001
Body fat rate, %	28.9(7.5)	26.8 (7.0)	28.5 (7.4)	29.7 (7.5)	30.8(7.5)	< 0.001	< 0.001
BAI	29.0(4.2)	28.2 (3.8)	28.8 (4.1)	29.3 (4.4)	29.6(4.4)	< 0.001	< 0.001
VAI	1.9(2.4)	0.7 (0.3)	1.2 (0.4)	1.8 (0.5)	3.8(4.0)	< 0.001	< 0.001
WHR	0.88 (0.07)	0.84 (0.07)	0.89 (0.06)	0.89 (0.06)	0.91 (0.06)	< 0.001	< 0.001
HbA1, %	5.9 (0.9)	5.6 (0.4)	5.7 (0.5)	5.9 (0.6)	6.4 (1.3)	< 0.001	< 0.001
Fasting glucose, mg/dL	97.7 (21.7)	89.5 (7.5)	93.4 (9.4)	97.0 (13.3)	111.0 (36.0)	< 0.001	< 0.001
Total cholesterol, mg/dL	197.3 (26.8)	188.1 (33.6)	195.8 (34.5)	200.9 (37.1)	204.5 (39.4)	< 0.001	< 0.001
Triglyceride, mg/dL	121.4 (100.9)	55.1 (11.0)	84.6 (11.3)	120.18 (18.3)	225.74 (153.3)	< 0.001	< 0.001
HDL-C, mg/dL	54.7 (13.7)	64.6 (13.6)	57.5 (12.3)	51.8 (10.9)	45.0 (9.5)	< 0.001	< 0.001
LDL-C, mg/dL	121.02 (32.4)	109.9 (28.4)	122.54 (29.9)	128.57 (32.7)	123.11 (35.3)	< 0.001	< 0.001
Albumin, g/dL	4.48 (0.2)	4.44 (0.2)	4.46 (0.2)	4.49 (0.2)	4.52 (0.2)	< 0.001	< 0.001
AST, U/L	26.2 (13.6)	24.4 (10.6)	25.3 (12.0)	26.4 (13.5)	28.5 (16.9)	< 0.001	< 0.001
ALT, U/L	24.2 (23.5)	18.8 (18.7)	21.8 (19.0)	25.5 (25.7)	30.8 (27.4)	< 0.001	< 0.001
Creatinine, mg/dL	0.7 (0.4)	0.7 (0.3)	0.7 (0.3)	0.8 (0.4)	0.8 (0.5)	< 0.001	< 0.001
Uric acid, mg/dL	5.4 (1.4)	4.8 (1.2)	5.3 (1.3)	5.6 (1.4)	6.0 (1.5)	< 0.001	< 0.001
THR	2.6 (3.2)	0.9 (0.3)	1.5 (0.4)	2.4 (0.7)	5.4(5.4)	< 0.001	< 0.001
Fatty liver, moderate or severe	3044 (15.4)	84 (1.7)	401 (8.1)	859 (17.4)	1700 (55.8)	< 0.001	< 0.001
Carotid plaques, moderate or severe	2115 (10.7)	326 (6.6)	47 (0.9)	577 (11.7)	739(34.9)	< 0.001	< 0.001

^a^ Data are presented as number (%) for categorical variables or mean (SD) for numerical variables. WHR is reported to two decimal places to maintain differentiation across quartiles that would be lost with single-digit rounding. ^b^ TyG index calculated as: ln[triglycerides(mg/dl) x blood glucose(mg/dl)/2]. Participants were divided into quartiles (Q1–Q4) based on their TyG index levels. ^c^ Differences were tested with chi-square for categorical variables and with analysis of covariance for numerical data. ^d^ Smoking: defined as current smokers, having smoked continuously for 6 months and being still smoking at the time of the interview. ^e^ Drinking: defined as having a drinking habit (150 c.c/week for 6 months) and being currently drinking alcohol. ^f^ Exercise: defined as exercising regularly (at least three times a week for 30 min or more). ^g^ Abdominal Obesity: WC ≥ 90 cm in men; ≥ 80 cm in women. ^h^ High blood pressure: SBP ≥ 130mmHg or DBP ≥ 85mmHg. ^i^ High fasting glucose: fasting glucose ≥ 100 mg/dL. ^j^ High triglyceride: triglyceride ≥ 150 mg/dL. ^k^ Low HDL-C: refer to high density lipoprotein cholesterol (HDL-C) < 40 mg/dL in men; <50 mg/dL in women.Abbreviations: TyG Index, triglyceride-glucose index; BMI, body mass index; BAI, body adiposity index; VAI, visceral adiposity index; WHR, waist-to-hip ratio; HbA1c, hemoglobin A1c; HDL-C, high-density lipoprotein cholesterol; LDL-C, low-density lipoprotein cholesterol; AST, aspartate aminotransferase; ALT, alanine aminotransferase; THR, triglyceride to HDL-C ratio

### Association between TyG index and cancer incidence

After excluding 1,783 participants diagnosed with cancer within the first year of enrollment, 148,809 participants were eligible for cancer tracking through the data linkage with TCR until December 2022. During a median follow-up of 5.7 years (IQR: 3.4–7.6), 4,467 individuals developed cancer. Table 3 summarizes the incidence of prevalent cancers across TyG index quartiles in this cancer-free population at one year post-enrollment. A significant association was observed between higher TyG index quartiles and increased risk of several cancer types. Detailed ICD-9 and ICD-10 codes for these cancers along with the incidence of a wider range of cancers across TyG quartiles, are provided in Supplementary Tables 1 and 2.

**Table 3:** Cancer incidence during follow-up across TyG index quartiles in individuals cancer-free one year post-enrollment in the Taiwan biobank, categorized by major organ systems

Types of Cancer^a^	New cancer cases in individuals cancer-free one year post-enrollment	TyG index^b^, Q1 (6.1–7.9)	TyG index, Q2 (8.0-8.3)	TyG index, Q3 (8.4–8.7)	TyG index, Q4 (8.8–13.1)	*P*_trend_	*P*_fdr_
	*N* = 148,809 (94,709 women)	*N* = 37,217 (29,361 women)	*N* = 37,174 (25,283 women)	*N* = 37,223 (22,100 women)	*N* = 37,195 (17,965 women)		
All cancers (total)	4,467 (3.0%)	941 (2.5%)	1,153 (3.1%)	1,176 (3.2%)	1,193 (3.2%)	< 0.001	< 0.001
Head and neck	174 (0.1%)	24 (0.1%)	38 (0.1%)	42 (0.1%)	69 (0.2%)	< 0.001	< 0.001
Eye, brain, and CNS	50 (0.03%)	12 (0.03%)	16 (0.04%)	13 (0.03%)	9 (0.02%)	0.449	0.593
Respiratory and intrathoracic organs	740 (0.50%)	156 (0.42%)	183 (0.49%)	209 (0.56%)	191 (0.51%)	0.031	0.091
Trachea/bronchus/lung^c^	689 (0.46%)	149 (0.40%)	168 (0.45%)	195 (0.52%)	176 (0.47%)	0.065	0.139
Digestive organs	1,092 (0.73%)	175 (0.47%)	251 (0.68%)	300 (0.81%)	366 (0.98%)	< 0.001	< 0.001
Colon/rectum^c^	559 (0.38%)	90 (0.24%)	125 (0.34%)	166 (0.45%)	178 (0.48%)	< 0.001	< 0.001
Genital organs	771 (0.52%)	151 (0.41%)	220 (0.59%)	199 (0.53%)	201 (0.54%)	0.037	0.105
Prostate, male	318 (0.59%)	44 (0.56%)	81 (0.68%)	92 (0.61%)	101 (0.53%)	0.321	0.421
Urinary tract	201 (0.13%)	27 (0.07%)	39 (0.10%)	54 (0.15%)	81 (0.22%)	< 0.001	< 0.001
Breast, female	1,090 (1.15%)	298 (1.01%)	299 (1.18%)	273 (1.24%)	220 (1.22%)	0.019	0.048
Thyroid and endocrine glands	272 (0.18%)	72 (0.19%)	79 (0.21%)	66 (0.18%)	53 (0.14%)	0.057	0.131
Skin	77 (0.05%)	18 (0.05%)	21 (0.06%)	19 (0.05%)	19 (0.05%)	0.959	0.982
Lymphoid and hematopoietic	205 (0.14%)	42 (0.11%)	58 (0.16%)	49 (0.13%)	56 (0.15%)	0.302	0.426

^a^ Data are presented as number (%). Percentages in parentheses represent cancer incidence proportions within each TyG quartile. All values are presented to two decimal places to preserve clarity and allow consistent comparison across cancer types, including those with low event rates (< 0.1%). For sex-specific cancers, the incidence proportions were calculated using the number of women (for breast cancer) or men (for prostate cancer) in each TyG quartile as the denominator, unlike other cancers where total quartile population was used. The correspondence of ICD-9-CM and ICD-10-CM codes for various cancer types is provided in supplementary Table 1. Certain organ systems, such as the retroperitoneum/peritoneum and bone/articular cartilage, were not included in the analysis due to the small number of malignant cases (20 cases and 4 cases, respectively, among 148,920 participants). Due to the lower cumulative incidence of some cancer types within certain organ systems, only the more common cancers from major organ systems are listed in this table, with detailed information in Supplementary Table 2. ^b^ TyG index calculated as: ln[triglycerides(mg/dl) x blood glucose(mg/dl)/2]. Participants were divided into quartiles (Q1–Q4) based on their TyG index levels. ^c^ Cancer of the trachea, bronchus, and lung comprises the majority of cancers in the respiratory and intrathoracic organs (689 out of 740 cases). Similarly, colorectal cancer represents more than half of the digestive system cancer cases (552 of 1092).Abbreviations: P_trend_, P value for trend; P_fdr_, false discovery rate-adjusted P trend value; CNS, central nervous system

The incidence of all cancers increased progressively across TyG index quartiles, rising from 2.53% in the lowest quartile (Q1) to 3.21% in the highest quartile (Q4) (P_trend_ < 0.001, P_fdr_ < 0.001), indicating a strong positive correlation between elevated TyG levels and overall cancer risk. Specific cancers, including head and neck, digestive system, colorectal, and urinary tract cancers, exhibited similar significant trends across TyG quartiles (P_trend_ < 0.001, P_fdr_ < 0.001). Colorectal cancer, representing over half of digestive system cancer cases, increased from 0.24% in Q1 to 0.48% in Q4. Despite a nominally significant unadjusted trend for breast cancer (P_trend_ = 0.019), the FDR-adjusted value (P_fdr_ = 0.048) was borderline, and the incidence did not show a consistent linear increase across TyG quartiles, suggesting a more complex or weaker relationship between IR and breast cancer risk.

**Fig. 2:**
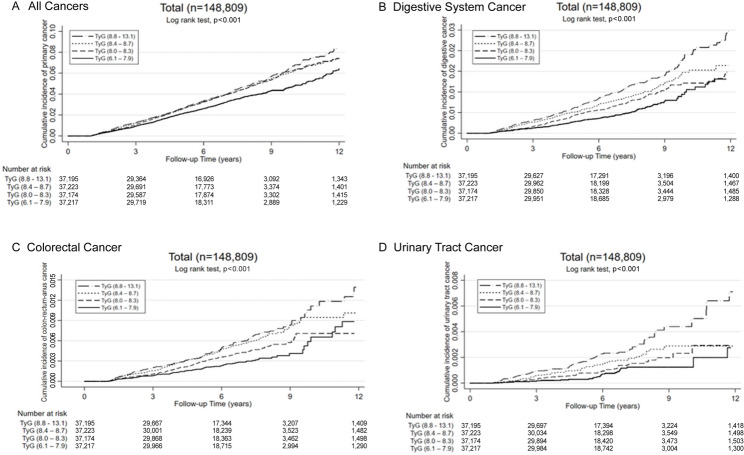
Cumulative incidence of specific types of cancer stratified by triglyceride-glucose (TyG) index quartiles in the Taiwan Biobank population. Cumulative incidence curves for specific cancer types are presented across TyG index quartiles in the Taiwan Biobank cohort (N = 148,809). A significant dose-response relationship was observed, with higher TyG index quartiles associated with increased cancer incidence (log-rank test, p < 0.001). **A**: Cumulative incidence of all cancers across TyG index quartile. **B**: Cumulative incidence of digestive system cancer across TyG index quartile. **C**: Cumulative incidence of colorectal cancer across TyG index quartile. **D**: Cumulative incidence of urinary tract cancer across TyG index quartile

Subsequent analyses focused on cancers with higher prevalence and stronger associations with the TyG index: all cancers (total), digestive system cancer, colorectal cancer (a major subset of digestive cancers), and urinary tract cancer (Table 3). As colorectal cancer accounts for over half of digestive system cancer cases, its inclusion in further analysis was well-justified. Fig. 2 presents cumulative incidence curves by TyG quartiles, revealing a clear dose-response relationship. Individuals in the highest TyG quartile exhibited significantly higher cumulative incidences for all cancers, digestive system cancer, colorectal cancer, and urinary tract cancer. The log-rank test (*P* < 0.001) confirmed the separation between curves, reinforcing the association between elevated TyG levels and increased cancer risk.

### Association between TyG index and cancer risk: multivariate Cox regression analysis and subgroup analysis

Multivariate Cox regression analysis of the TyG index and the risk of all cancers, digestive system cancer, colorectal cancer, and urinary tract cancer, adjusted for sex, age, BMI, habits of cigarette smoking, alcohol drinking, and exercise, provides a clearer evaluation of its independent impact on cancer risk.

**Fig. 3:**
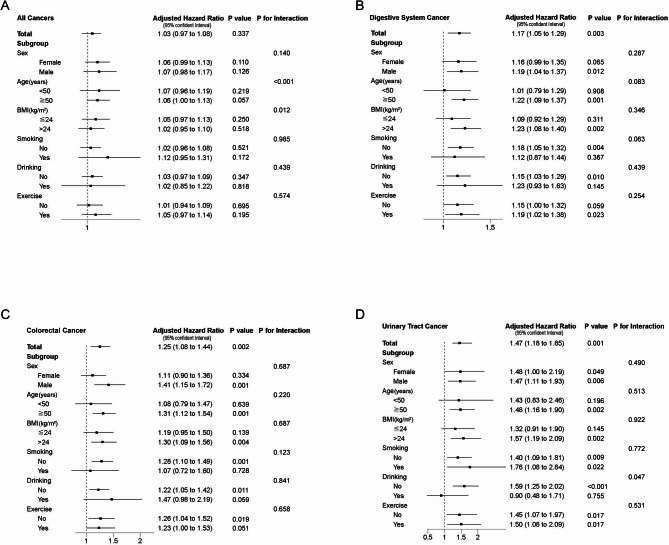
Impact of triglyceride-glucose (TyG) index on cancer risk: Adjusted Hazard Ratios (aHRs) for all, digestive system, colorectal, and urinary tract cancers, and stratified by demographic and lifestyle factors. Panels A–D show aHRs with 95% confidence intervals (CIs) for the associations between TyG index (treated as a continuous variable) and cancer risk, further stratified by sex, age (50 years), BMI (24 kg/m2), smoking, drinking, or exercise habits to explore the relationship between TyG index (continuous variable) and different cancers. **A**: aHR for all cancer risk associated with the TyG index. **B**: aHR for digestive system cancer risk associated with the TyG index. **C**: aHR for colorectal cancer risk associated with the TyG index. **D**: aHR for urinary tract cancer risk associated with the TyG index. All aHRs were adjusted for sex, age, BMI, smoking, drinking, and exercise habits (except stratification variables)

In addition, interaction testing was conducted to examine whether the association between TyG index and cancer risk varied across demographic and behavioral subgroups. Significant interactions were observed for all cancers by age (*P* < 0.001) and BMI (*P* = 0.012), and for urinary tract cancer by drinking status (*P* = 0.047). No other interactions reached statistical significance. Full results are presented in Supplementary Tables 3 and summarized alongside adjusted HRs in Fig. 3.

#### All cancers

As shown in Fig. 3A, no overall significant association was observed between the TyG index and overall cancer risk (aHR: 1.03, 95% CI: 0.97–1.08, *P* = 0.34). But interaction analyses indicated significant effect modification by age (*P* < 0.001) and BMI (*P* = 0.012), suggesting that the relationship may vary across subgroups.

#### Digestive system and colorectal cancer

Fig. 3B and C demonstrate a significant independent association between each one-unit increase in the TyG index and an elevated risk of digestive system cancer (aHR: 1.17, 95% CI: 1.05–1.29, *P* = 0.003) and colorectal cancer (aHR: 1.25, 95% CI: 1.08–1.44, *p* = 0.002). Although subgroup analyses indicated numerically higher risks of digestive system and colorectal cancers among males, older individuals, those with overweight or obesity, and non-smokers, none of the interaction terms reached statistical significance.

#### Urinary tract cancer

Fig. 3D shows a significant independent association between the TyG index and urinary tract cancer, with each one-unit increase in the TyG index corresponding to a 47% higher risk of urinary tract cancer (aHR: 1.47, 95% CI: 1.18–1.85, *P* < 0.01). This risk was especially pronounced among males (aHR: 1.47, 95% CI: 1.11–1.93, *P* < 0.01), participants aged 50 or older (aHR: 1.48, 95% CI: 1.16–1.90, *P* < 0.01), and those with a BMI over 24 kg/m² (aHR: 1.57, 95% CI: 1.19–2.09, *P* < 0.01). Stratified by smoking status, the TyG index was significantly associated with urinary tract cancer in both smokers (aHR: 1.76, 95% CI: 1.08–2.84, *p* = 0.02) and non-smokers (aHR: 1.40, 95% CI: 1.09–1.81, *p* < 0.01). Although the point estimate appeared higher in smokers, the interaction test (*P* = 0.77) did not support a statistically significant difference in effect between the groups. Additionally, interaction analysis revealed a significant effect modification by drinking status (*P* = 0.047), suggesting that alcohol consumption may influence the relationship between TyG index and urinary tract cancer risk.

## Discussion

This longitudinal cohort study examined the relationship between the TyG index, a validated marker of IR, and cancer incidence in a Taiwanese population of 150,592 participants over a decade of follow-up. Elevated TyG index levels were significantly associated with increased risks of digestive system, colorectal, and urinary tract cancers, even after adjusting for demographic and lifestyle factors, including age, sex, BMI, smoking, alcohol consumption, and exercise habits. Additionally, participants in higher TyG quartiles exhibited adverse metabolic profiles, characterized by greater adiposity, disrupted glucose and lipid metabolism at baseline and follow-up. A higher TyG quartile also accompanied a higher prevalence of fatty liver and carotid plaques. These demonstrate the TyG index’s utility as an indicator of persistent metabolic dysfunction.

The dose-response relationship between TyG index levels and cumulative cancer incidence supports the role of IR in oncogenesis of digestive system cancer, colorectal cancer and urinary tract cancer [35, 36]. Our study builds on prior evidence linking IR to cancer risk by utilizing the TyG index, a simple and broadly applicable surrogate marker of IR, to evaluate its association with multiple cancer types in a large community-based cohort. While previous research has largely focused on individual cancers or smaller populations, our findings offer broader insights into the epidemiologic and biological relevance of IR in cancer development.

IR, as a central metabolic consequence of obesity and visceral adiposity, is thought to mediate the well-established association between excess adiposity and increased cancer risk. Hyperinsulinemia, a hallmark of IR, activates the insulin-like growth factor (IGF) signaling axis, enhancing cell proliferation and inhibiting apoptosis [4, 37]. Moreover, IR is closely linked to chronic low-grade inflammation and oxidative stress, both of which can contribute to DNA damage, genomic instability, and oncogenic transformation [38–40]. Emerging evidence also suggests that IR may interact with tissue-specific oncogenic pathways, potentially influencing the initiation or progression of certain cancers in a context-dependent manner. These mechanisms are particularly relevant in metabolically active or stress-sensitive tissues such as the colorectal mucosa, liver, pancreas, and urothelium [38–41]. The colorectal mucosa, with its high turnover rate, is susceptible to hyperproliferation; the liver and pancreas are exposed to metabolic overload; and the bladder epithelium may be vulnerable to oxidative stress–related injury. These biologic pathways may help explain why we observed stronger associations between the TyG index and cancers of the digestive and urinary systems, compared to a more modest association with overall cancer risk. The attenuated signal in the overall cancer outcome may reflect the heterogeneity of cancer etiologies, where metabolically unrelated cancers dilute the overall effect size. In addition, age and BMI demonstrated statistically significant interaction effects in the context of overall cancer risk, suggesting that the association between IR and cancer development may be modified by these factors. These findings underscore the relevance of demographic and metabolic profiles in shaping cancer susceptibility and highlight the importance of stratified approaches in risk prediction. Further studies are needed to elucidate the molecular mechanisms linking IR to carcinogenesis and to enhance the translational and clinical utility of IR-based risk stratification strategies.

The association between elevated TyG index levels and urinary tract cancer was particularly pronounced, with the highest cumulative incidence observed in the top quartile (Q4), suggesting that the TyG index may serve as a useful marker for identifying risk, even in less common malignancies. Tobacco smoking has long been recognized as a major risk factor for bladder cancer, as established in prior epidemiologic literature [42]. Recent genetic evidence also links smoking to increased risk of MetS [43]. This supports the idea that smoking and IR may act together in cancer development. In our stratified analysis, the association between TyG index and urinary tract cancer appeared stronger among smokers (aHR: 1.76, 95% CI: 1.08–2.84) than non-smokers (aHR: 1.40, 95% CI: 1.09–1.81). However, the interaction test yielded a P value of 0.77, indicating no statistically significant effect modification. These findings suggest that although smoking has been associated with both urothelial carcinogenesis and metabolic dysfunction, it does not appear to influence the relationship between IR and urinary tract cancer risk in this cohort.

Previous studies have established that IR is closely linked to key features of MetS, including visceral adiposity, impaired glucose regulation, dyslipidemia, and central obesity. Each of these components contributes to increased risk for cardiometabolic diseases such as non-alcoholic fatty liver disease, endothelial dysfunction, and atherosclerosis [18, 44]. In line with these findings, our study demonstrated that individuals in higher TyG index quartiles exhibited consistently adverse metabolic profiles, including abdominal obesity, elevated fasting glucose, high TG levels, and low HDL-C, observed both at baseline and during follow-up. Furthermore, these individuals showed greater visceral fat accumulation and a higher prevalence of fatty liver and carotid atherosclerosis, suggesting early signs of subclinical organ damage. Such metabolic alterations may act as intermediate phenotypes linking IR to increased cancer susceptibility. As a validated surrogate marker of IR, the TyG index not only captures the severity of metabolic dysfunction but also reflects its persistence over time, as supported by a strong correlation between baseline and follow-up values (*r* = 0.75; Supplementary Fig. 1). Collectively, these findings suggest that the TyG index may serve as a unifying surrogate marker that links IR, MetS-related dysfunction, and increased cancer risk through shared biological mechanisms.

Although adjusted hazard ratios appeared higher in men than in women for several cancer types, the statistical tests for interaction between sex and the TyG index were not significant. This suggests that while sex-based trends may exist, they should be interpreted cautiously and warrant further investigation. Nonetheless, given prior evidence that visceral adiposity, which is more prevalent in men, contributes to metabolic dysfunction and cancer development [45], incorporating sex-specific factors into future risk stratification models may still be of clinical interest.

Our study has several strengths. Utilizing the TWB and its linkage to the TCR allowed comprehensive long-term participant tracking, minimizing follow-up bias. The large sample size of over 150,000 participants and the use of standardized data collection procedures provided substantial statistical power to detect significant associations between the TyG index and cancer risk.

However, some limitations should be considered. First, the study was conducted exclusively within a Taiwanese population, which may limit the generalizability of the findings to other ethnic or geographic groups. Genetic, lifestyle, and environmental differences may influence the association between metabolic markers, including the TyG index, and disease outcomes in other populations. While the absolute TyG index levels and quartile boundaries vary across populations, as reported in studies from Korea and Spain [46, 47], individuals in higher TyG quartiles consistently exhibited greater cardiometabolic risk. Similarly, in our Taiwanese cohort, those in higher TyG quartiles had worse metabolic profiles and an elevated risk of cancer. This consistency across populations suggests that, despite inter-population differences in absolute TyG values, relative TyG stratification remains a meaningful approach for risk assessment. Population-specific risk patterns and differences in body composition may also influence the interpretation of other metabolic indicators, such as BMI. In this study, we used BMI categories defined by Taiwan’s Ministry of Health and Welfare to reflect local clinical practice and risk classification. However, we acknowledge that adopting WHO-recommended thresholds (e.g., BMI ≥ 25 kg/m² for overweight) would enhance comparability with international studies. Future research should consider incorporating both classification systems to improve cross-population interpretation and to determine optimal BMI cut-offs tailored to different ethnic groups. Second, although we adjusted for key confounders, such as age, BMI, smoking, alcohol consumption, and exercise, residual confounding from unmeasured factors, such as diet, physical activity intensity, socioeconomic status, education level, and family history of cancer could still influence the observed associations. Third, our analysis primarily relied on baseline TyG index measurements, which may not fully reflect changes in IR over time. To address this limitation, we assessed the correlation between baseline and follow-up TyG values in individuals who completed follow-up check-ups. The strong correlation observed suggests that individuals with higher baseline TyG levels tended to maintain elevated levels over time. Moreover, participants with higher follow-up TyG levels showed adverse metabolic profiles, consistent with baseline patterns. This supports the use of baseline TyG as a reliable predictor of persistent IR, adverse metabolic outcomes, and long-term cancer risk. Fourth, while the median follow-up duration of 5.7 years enables the evaluation of mid-term cancer risk, it may be insufficient to fully capture malignancies with longer latency periods. Although we excluded cancers diagnosed within the first year to mitigate reverse causality, it remains possible that some latent cancers were still present but undetected in the early years of follow-up. Due to current restrictions on dataset access, further sensitivity analyses such as applying a longer exclusion window (e.g., three years) could not be conducted in the present study. Despite this limitation, our Kaplan–Meier survival curves provide some indirect support against early reverse causality. Cumulative incidence curves became increasingly separated after the third year of follow-up, especially for site-specific cancers such as digestive system and urinary tract cancers. The highest TyG quartiles demonstrated steeper slopes, indicating a faster accumulation of cancer events over time. These observed patterns support a sustained association between elevated TyG index levels and cancer risk beyond the early follow-up period. As the Taiwan Biobank continues to accrue longitudinal data, future research could incorporate repeated measurements of the TyG index and extended follow-up to better characterize the dynamic relationship between IR and cancer development, particularly for long-latency cancers.

## Conclusion

Elevated TyG index levels, a practical and accessible surrogate marker of IR, are associated with a higher risk of digestive system, colorectal, and urinary tract cancers. These findings demonstrate the potential utility of the TyG index in identifying individuals at increased metabolic risk and guiding targeted prevention strategies. Further research is needed to assess whether reducing IR can lower cancer incidence and to elucidate the metabolic pathways linking IR to cancer. Incorporating the TyG index into routine metabolic assessments may improve cancer risk stratification and enhance prevention efforts.

## Electronic supplementary material

Below is the link to the electronic supplementary material.

Supplementary Material 1

## Data Availability

The data of the findings of this study are available from the corresponding author upon request.

## Abbreviations

aHR: Adjusted hazard ratio
ALT: Alanine aminotransferase
ANOVA: Analysis of variance
AST: Aspartate aminotransferase
BAI: Body adiposity index
BMI: Body mass index
CI: Confidence interval
DBP: Diastolic blood pressure
FDR: False discovery rate
FPG: Fasting plasma glucose
HbA1c: Hemoglobin A1c
HC: Hip circumference
HDL-C: High-density lipoprotein cholesterol
HOMA-IR: Homeostasis model assessment of insulin resistance
HR: Hazard ratio
ICD: International classification of diseases
IDF: International diabetes federation
IGF: Insulin-like growth factor
IR: Insulin resistance
LDL-C: Low-density lipoprotein cholesterol
MetS: Metabolic syndrome
NHIRD: National health insurance research database
P_trend_: Trend p-value
Q: Quartile
SBP: Systolic blood pressure
SD: Standard deviation
TCR: Taiwan cancer registry
TG: Triglycerides
THR: Triglyceride to high-density lipoprotein cholesterol ratio
TWB: Taiwan biobank
TyG: Triglyceride-glucose
UA: Uric acid
VAI: Visceral adiposity index
WC: Waist circumference
WHR: Waist-to-hip ratio
