# Supplementary material for: Triglyceride-glucose index and cancer risk: a prospective cohort study in Taiwan
Source: Diabetol Metab Syndr. 2025 Jul 18;17:283. doi: 10.1186/s13098-025-01768-8 (PMC12275392; doi:10.1186/s13098-025-01768-8)
Supplement: Supplementary file 1 — Supplementary Material 1 [file 13098_2025_1768_MOESM1_ESM.docx]

**Supplementary Data**

Supplementary Figure1: Correlation of TyG index at baseline evaluation and TyG index at follow-up visits


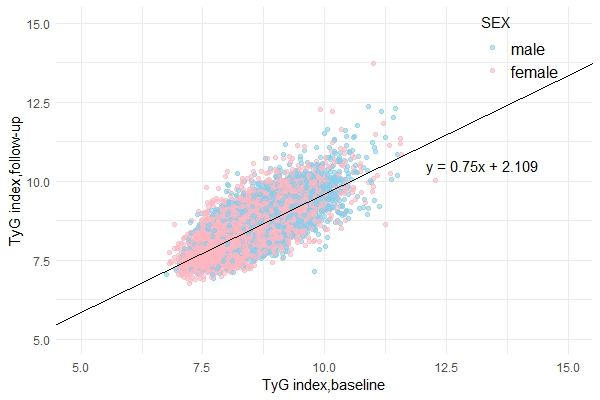


Correlation: 0.75

Supplementary Table 1: Correspondence of ICD-9-CM and ICD-10-CM Codes for Various Cancer Types

| **Type of cancer** | **ICD-10** | **ICD-9** |
| --- | --- | --- |
| Primary | C00-C75 | 140-194 |
| Head/neck cancer | C00-C06, C09-C14, C30-C31 | 140-141, 143-149, 160-161 |
| Eye, brain, and CNS | C69-C72 | 190-192 |
| Respiratory and intrathoracic organs | C30-C39 | 160-165 |
| Trachea/bronchus/lung | C33-C34 | 162 |
| Digestive system (including gastrointestinal tract from esophagus to rectum, and biliary tract including liver, bile duct, gall bladder and pancreas) | C15-C26 | 150-159 |
| Colon/rectum/anus cancer | C18-C21 | 153、1540、1541、1542、1543、1548 |
| Genital organs | C51-C58, C60-C63 | 179-187 |
| Prostatic cancer | C61 | 185 |
| Urinary tract | C64-C68 | 188-189 |
| Breast cancer | C50 | 174-175 |
| Thyroid and endocrine glands | C73-C75 | 193-194 |
| Skin | C43-C44 | 172-173 |
| Lymphoid and hematopoietic | C81-C96 | 200-208 |

Supplementary Table 2: Cumulative Incidence of Cancer across TyG Quartiles in the Study Population

|  |  |  | New cancer cases in individuals cancer-free one year post-enrollment |  |  |  |  |  |  |
| --- | --- | --- | --- | --- | --- | --- | --- | --- | --- |
| **Types of Cance** | **ICD-10** | **ICD-9** |  | TyG(Q1) | TyG(Q2) | TyG(Q3) | TyG(Q4) |  |  |
|  |  |  | N=148,809 | N=37,217 | N=37,174 | N=37,223 | N=37,195 | P_trend_ | FDR P_trend_ |
|  |  |  | 94,709 women | 29,361women | 25,283 women | 22,100 women | 17,965 women |  |  |
|  |  |  | 54,100 men | 7,856 men | 11,891 men | 15,123 men | 19,230 men |  |  |
| **All cancers** | **C00-C75** | **140-194** | 4,467 (3.00%) | 941 (2.53%) | 1,153 (3.10%) | 1,176 (3.16%) | 1,193 (3.21%) | <0.001 | <0.001 |
| **Head/neck cancer** | **C00-C06, C09-C14, C30-C31** | **140-141, 143-149, 160-161** | 174 (0.12%) | 24 (0.06%) | 38 (0.10%) | 42 (0.11%) | 69 (0.19%) | <0.001 | <0.001 |
| **Lip, oral cavity and pharynx** | **C00-C14** | **140-149** | 178 (0.12%) | 26 (0.07%) | 41 (0.11%) | 45 (0.12%) | 65 (0.17%) | <0.001 | <0.001 |
| **Oral, oropharyngeal, and hypopharyngeal cancers** | **C00-C06, C09-C10, C12-C14** | **140-141, 143-146, 148-149** | 101 (0.07%) | 10 (0.03%) | 22 (0.06%) | 26 (0.07%) | 43 (0.12%) | <0.001 | <0.001 |
| **Nasopharynx** | **C11** | **147** | 63 (0.04%) | 13 (0.03%) | 13 (0.03%) | 14 (0.04%) | 22 (0.06%) | 0.113 | 0.207 |
| **Digestive organs** | **C15-C26** | **150-159** | 1,092 (0.73%) | 175 (0.47%) | 251 (0.68%) | 300 (0.81%) | 366 (0.98%) | <0.001 | <0.001 |
| **Esophageal cancer** | **C15** | **150** | 30 (0.02%) | 5 (0.01%) | 5 (0.01%) | 4 (0.01%) | 16 (0.04%) | 0.011 | 0.038 |
| **Gastric cancer** | **C16** | **151** | 126 (0.08%) | 23 (0.06%) | 32 (0.09%) | 34 (0.09%) | 37 (0.10%) | 0.080 | 0.157 |
| **Small intestine cancer** | **C17** | **152** | 15 (0.01%) | 5 | | 10 | | 0.021 | 0.069 |
| **Colon/rectum cancer** | **C18-C21** | **153、1540、1541、1542、1543、1548** | 559 (0.38%) | 90 (0.24%) | 125 (0.34%) | 166 (0.45%) | 178 (0.48%) | <0.001 | <0.001 |
| **Liver cancer** | **C22** | **155** | 245 (0.16%) | 41 (0.11%) | 57 (0.15%) | 63 (0.17%) | 84 (0.23%) | <0.001 | <0.001 |
| **Pancreatic cancer** | **C25** | **157** | 116 (0.08%) | 15 (0.04%) | 30 (0.08%) | 26 (0.07%) | 45 (0.12%) | <0.001 | 0.002 |
| **Gallbladder & extrahepatic bile duct cancer** | **C23-C24** | **156** | 26 (0.02%) | 3 (0.01%) | 4 (0.01%) | 10 (0.03%) | 9 (0.02%) | 0.039 | 0.105 |
| **Respiratory and intrathoracic organs** | **C30-C39** | **160-165** | 740 (0.50%) | 156 (0.42%) | 183 (0.49%) | 209 (0.56%) | 191 (0.51%) | 0.031 | 0.091 |
| **Trachea/bronchus/lung** | **C33-C34** | **162** | 689 (0.46%) | 149 (0.40%) | 168 (0.45%) | 195 (0.52%) | 176 (0.47%) | 0.065 | 0.139 |
| **Thymus cancer** | **C37** | **164** | 26 (0.02%) | 10 (0.01%) | | 9 (0.02%) | 7 (0.02%) | 0.164 | 0.273 |
| **Thymus, heart and mediastinum** | **C37-C38.3, C38.8** | **164** | 31 (0.02%) | 5 (0.01%) | 9 (0.02%) | 10 (0.03%) | 7 (0.02%) | 0.574 | 0.712 |
| **Skin** | **C43-C44** | **172-173** | 77 (0.05%) | 18 (0.05%) | 21 (0.06%) | 19 (0.05%) | 19 (0.05%) | 0.959 | 0.982 |
| **Breast cancer, female** | **C50** | **174-175** | 1,090 (1.15%) | 298 (1.01%) | 299 (1.18%) | 273 (1.24%) | 220 (1.22%) | 0.019 | 0.048 |
| **Genital organs** | **C51-C58, C60-C63** | **179-187** | 771 (0.52%) | 151 (0.41%) | 220 (0.59%) | 199 (0.53%) | 201 (0.54%) | 0.037 | 0.105 |
| **Prostatic cancer, male** | **C61** | **185** | 318 (0.59%) | 44 (0.56%) | 81 (0.68%) | 92 (0.61%) | 101 (0.53%) | 0.321 | 0.421 |
| **Urinary tract** | **C64-C68** | **188-189** | 201 (0.13%) | 27 (0.07%) | 39 (0.10%) | 54 (0.15%) | 81 (0.22%) | <0.001 | <0.001 |
| **Kidney cancer** | **C64** | **189.0** | 50 (0.03%) | 10 (0.03%) | 10 (0.03%) | 9 (0.02%) | 21 (0.06%) | 0.045 | 0.111 |
| **Renal pelvis, ureter, and others** | **C65-C66, C68** | **189 (except 189.0)** | 68 (0.05%) | 8 (0.02%) | 23 (0.06%) | 13 (0.03%) | 24 (0.06%) | 0.041 | 0.105 |
| **Bladder cancer** | **C67** | **188** | 97 (0.07%) | 10 (0.03%) | 12 (0.03%) | 35 (0.09%) | 40 (0.11%) | <0.001 | <0.001 |
| **Eye, brain, and CNS** | **C69-C72** | **190-192** | 50 (0.03%) | 12 (0.03%) | 16 (0.04%) | 13 (0.03%) | 9 (0.02%) | 0.449 | 0.593 |
| **CNS** | **C70-C72** | **191-192** | 47 (0.03%) | 11 (0.03%) | 15 (0.04%) | 12 (0.03%) | 9 (0.02%) | 0.558 | 0.712 |
| **Brain cancer** | **C71** | **191** | 44 (0.03%) | 10 (0.03%) | 14 (0.04%) | 12 (0.03%) | 8 (0.02%) | 0.590 | 0.717 |
| **Thyroid and endocrine glands** | **C73-C75** | **193-194** | 272 (0.18%) | 72 (0.19%) | 79 (0.21%) | 66 (0.18%) | 53 (0.14%) | 0.057 | 0.131 |
| **Thyroid cancer** | **C73** | **193** | 268 (0.18%) | 69 (0.19%) | 79 (0.21%) | 65 (0.17%) | 53 (0.14%) | 0.089 | 0.168 |
| **Lymphoid and hematopoietic** | **C81-C96** | **200-208** | 205 (0.14%) | 42 (0.11%) | 58 (0.16%) | 49 (0.13%) | 56 (0.15%) | 0.302 | 0.426 |
| **Lymphoma** | **C81-C88** | **200, 201, 202** | 120 (0.08%) | 26 (0.07%) | 37 (0.10%) | 26 (0.07%) | 31 (0.08%) | 0.870 | 0.947 |
| **Non-Hodgkin lymphoma** | **C82-C85** | **200, 202-203** | 115 (0.08%) | 24 (0.06%) | 37 (0.10%) | 26 (0.07%) | 28 (0.08%) | 0.966 | 0.982 |
| **Bone & articular cartilage** | **C40-C41** | **170** | 4 (0.00%) | - | - | - | - | - | - |
| **Retroperitoneum & peritoneum** | **C48** | **158** | 20 (0.01%) | 6 (0.02%) | 5 (0.01%) | 6 (0.02%) | 3 (0.01%) | 0.426 | 0.586 |

Supplementary Table 3: Interaction P Values from Cox Regression Models Examining the Association between TyG Index and Cancer Risk across Subgroups

| Covariate | All cancers | Digestive system cancer | Colorectal cancer | Urinary tract cancer |
| --- | --- | --- | --- | --- |
| Sex | 0.140 | 0.083 | 0.687 | 0.490 |
| Age | <0.001 | 0.287 | 0.220 | 0.513 |
| BMI | 0.012 | 0.346 | 0.687 | 0.923 |
| Smoking | 0.985 | 0.063 | 0.123 | 0.772 |
| Drinking | 0.439 | 0.439 | 0.841 | 0.047 |
| Exercise | 0.574 | 0.254 | 0.658 | 0.531 |
